# Diverse biological characteristics and varied virulence of H7N9 from Wave 5

**DOI:** 10.1080/22221751.2018.1560234

**Published:** 2019-01-16

**Authors:** Linlin Bao, Yuhai Bi, Gary Wong, Wenbao Qi, Fengdi Li, Qi Lv, Liang Wang, Fei Liu, Yang Yang, Cheng Zhang, William J. Liu, Chuansong Quan, Weixin Jia, Yingxia Liu, Wenjun Liu, Ming Liao, George F. Gao, Chuan Qin

**Affiliations:** a Institute of Laboratory Animal Sciences, Chinese Academy of Medical Sciences (CAMS) & Comparative Medicine Center, Peking Union Medical Collage (PUMC); Key Laboratory of Human Disease Comparative Medicine, Ministry of Health, Beijing Key Laboratory for Animal Models of Emerging and Reemerging Infectious, Beijing, People’s Republic of China; b Shenzhen Key Laboratory of Pathogen and Immunity, Guangdong Key Laboratory for Diagnosis and Treatment of Emerging Infectious Diseases, State Key Discipline of Infectious Disease, Second Hospital Affiliated to Southern University of Science and Technology, Shenzhen Third People’s Hospital, People’s Republic of China; c CAS Key Laboratory of Pathogenic Microbiology and Immunology, Collaborative Innovation Center for Diagnosis and Treatment of Infectious Disease, Institute of Microbiology, Center for Influenza Research and Early-warning (CASCIRE), Chinese Academy of Sciences, Beijing, People’s Republic of China; d Département de microbiologie-infectiologie et d'immunologie, Université Laval, Québec City, Canada; e National and Regional Joint Engineering Laboratory for Medicament of Zoonoses Prevention and Control, College of Veterinary Medicine, South China Agricultural University, Guangzhou, People’s Republic of China; f National Institute for Viral Disease Control and Prevention, Chinese Center for Disease Control and Prevention (China CDC), Beijing, People’s Republic of China

**Keywords:** H7N9, influenza, receptor, ferret, pathogenicity, transmissibility

## Abstract

There was a substantial increase with infections of H7N9 avian influenza virus (AIV) in humans during Wave 5 (2016-2017). To investigate whether H7N9 had become more infectious/transmissible and pathogenic overall, we characterized the receptor binding and experimentally infected ferrets with highly pathogenic (HP)- and low pathogenic (LP)-H7N9 isolates selected from Wave 5, and compared their pathogenicity and transmissibility with a Wave 1 isolate from 2013. Studies show that A/Anhui/1/2013 (LP) and A/Chicken/Heyuan/16876/2016 (HP) were highly virulent in ferrets, A/Guangdong/Th008/2017 (HP) and A/Chicken/Huizhou/HZ-3/2017 (HP) had moderate virulence and A/Shenzhen/Th001/2016 (LP) was of low virulence in ferrets. Transmission was observed only in ferrets infected with A/Anhui/1/2013 and A/Chicken/Heyuan/16876/2016, consistent with the idea that sicker ferrets had a higher probability to transmit virus to naive animals. Given the Varied virulence and transmissibility observed in circulating H7N9 viruses from Wave 5, we conclude that the current public health risk of H7N9 has not substantially increased compared to 2013 and the circulating viruses are quite diverse.

## Introduction

H7N9 avian influenza viruses (AIVs) have caused six infection waves in China since 2013. A total of 1567 human cases (three cases in Wave 6, 2017-2018) were reported, with a case fatality rate of 39.2% (615 deaths, as of 5 September 2018) according to the World Health Organization (WHO) [1]. The highest number of cases was reported during Wave 5 in 2016-2017, and the geographical distribution was more widespread compared to previous waves [2]. This suggests that H7N9 may have evolved to become more infectious and/or transmissible, or possibly more virulent.

Previous H7N9 AIVs since 2013 were classified as low pathogenic (LP) in chickens and have limited transmissibility in ferrets [3–6]. During Wave 5, highly pathogenic (HP)-H7N9 variants emerged, in which isolates possessed an insertion of 2–3 additional basic amino acid residues at the haemagglutinin (HA) cleavage site (CS) [2,7–9]. Nearly all reported HP-H7N9 variants to date possess the Q226 residue in the HA protein [2], suggesting these viruses preferentially bind avian-type receptors (α2-3-SA) [10–13]. A recent study showed that an HP-H7N9 isolate preferentially bound avian-type receptors but was able to transmit among ferrets [14]. In contrast, studies on another HP-H7N9 isolate showed that the virus preferentially bound human-type receptors but did not transmit effectively in ferrets [15].

Completely different receptor binding and transmission characteristics were shown with these two wild-type HP-H7N9 isolates, and in Wave 5, infections with LP-H7N9 were still prominent despite the emergence of HP variants [2]. To investigate the exact public health risk posed by circulating H7N9 viruses, we studied the receptor-binding capability, pathogenicity and transmissibility of three HP- and one LP-H7N9 isolates from Wave 5.

## Results

### Receptor binding properties

**Figure 1.**
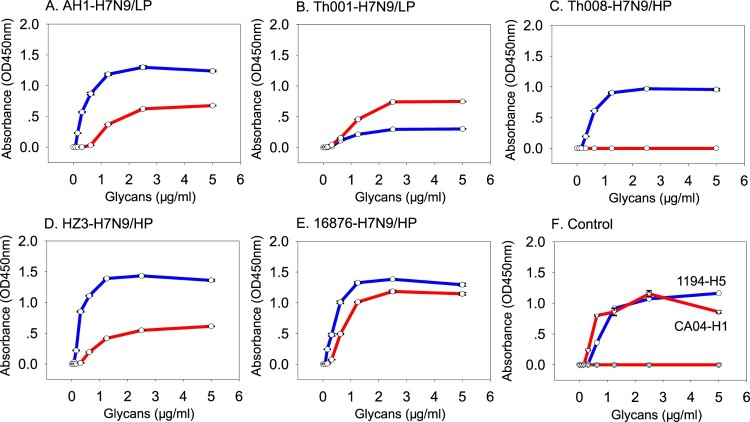
Receptor binding properties of the H7N9 viruses. Receptor binding properties of H7N9 to human (α2-6-SA) or avian (α2-3-SA) receptors were tested using the solid-phase direct binding assay with 6′SLNLN and 3′SLNLN. Red and blue represents human- and avian-origin receptors, respectively.

Receptor binding abilities of the HP- and LP-H7N9 in Wave 5 were tested using the human- (α2-6-SA) and avian- (α2-3-SA) type receptors. The ancestor A/Anhui/1/2013 (LP) was found to bind both receptor types, with higher affinity for avian than human (Figure 1(A) and Table 1). A/Shenzhen/Th001/2016 (LP) also displayed binding to both human and avian receptors. In contrast, A/Shenzhen/Th001/2016 (LP) preferentially bound human receptors, but the avidity was poor and similar to that of A/Anhui/1/2013 (LP) (Figure 1(B) and Table 1).

**Table 1.** Biological and disease characteristics of LP- and HP-H7N9 viruses in Wave 5.

Viruses	Receptor binding profile^a^		Pathogenicity in ferrets				Replication in nasal cavity		Transmissibility in ferrets	
	α2-3-SA	α2-6-SA	Proportion of animals with clinical signs	Peak clinical scores^b^ (AVG ± SD)	Disease duration (days) (AVG ± SD)	Proportion of animals with ≥7% weight loss	Peak virus titres (PFU/ml) (AVG ± SD)	Virus shedding duration (days)^c^ (AVG ± SD)	Virus detected	Seroconversion
**A/Anhui/1/2013 (LP)**	+++	++	3/3	2.0 ± 1.0	7.0 ± 3.6	2/3	9.4E3 ± 1.0E4	5.7 ± 1.2	1/3	2/3
**A/Shenzhen/Th001/2016 (LP)**	+	++	3/3	1.0 ± 0.0	2.0 ± 1.0	0/3	2.0E3 ± 3.0E2	5.0 ± 0.0	0/3	0/3
**A/Guangdong/Th008/2017 (HP)**	+++	–	3/3	1.7 ± 1.2	3.0 ± 1.0	1/3	3.5E2 ± 2.1E2	3.7 ± 0.6	0/3	0/3
**A/Chicken/Huizhou/HZ-3/2017 (HP)**	+++	++	3/3	1.7 ± 0.6	2.0 ± 1.7	1/3	8.2E2 ± 1.1E3	3.7 ± 1.5	0/3	0/3
**A/Chicken/Heyuan/16876/2016 (HP)**	+++	+++	3/3	2.0 ± 1.0	8.7 ± 3.8	2/3	1.8E5 ± 2.5E5	8.3 ± 1.2	1/3	1/3

^a^Receptor binding OD values below 0.5 were marked as “+”, between 0.5 and 1.0 were “++”, and between 1.0 and 1.5 were shown as “+++”. The negative result was shown as “−”.

^b^Clinical scores are the sum of two parts. Part one is nasal symptoms: no symptoms (0), nasal rattling or sneezing (1), nasal discharge on external nasal cavity (2) and mouth breathing (3). Part 2 is activity level: playful (0), not initiating play (1), alert but not playful (2) and not playful, not alert (3).

^c^From first day to last day of virus detection.

Interestingly, the receptor-binding properties of HP-H7N9 isolates were variable. A/Guangdong/Th008/2017 (HP) only bound avian receptors (Figure 1(C) and Table 1). A/Chicken/Huizhou/HZ-3/2017 (HP) and A/Chicken/Heyuan/16876/2016 (HP) bound both human and avian receptors (Figure 1(D–E) and Table 1), with higher affinity to avian receptors for both isolates (Figure 1(D–E) and Table 1). As expected, controls 1194-H5N1 and CA04-H1N1 only bound avian and human receptors, respectively (Figure 1(F)).

### Pathogenicity in ferrets

Ferrets (*n* = 3 per group) were used to study the pathogenesis of the various H7N9 isolates. Sneezing, ruffled fur and decreased appetite were observed in the infected ferrets, but their activity levels did not change. Weight loss was observed to different degrees in all groups (Figure S1 and Table 1). For isolates A/Anhui/1/2013 (LP) and A/Chicken/Heyuan/16876/2016 (HP), 66% (2/3) ferrets had over 7% body weight loss. For A/Guangdong/Th008/2017 (HP) and A/Chicken/Huizhou/HZ-3/2017 (HP), 33% (1/3) ferrets had over 7% body weight loss, and for A/Shenzhen/Th001/2016 (LP) it was 0% (0/3).

**Figure 2.**
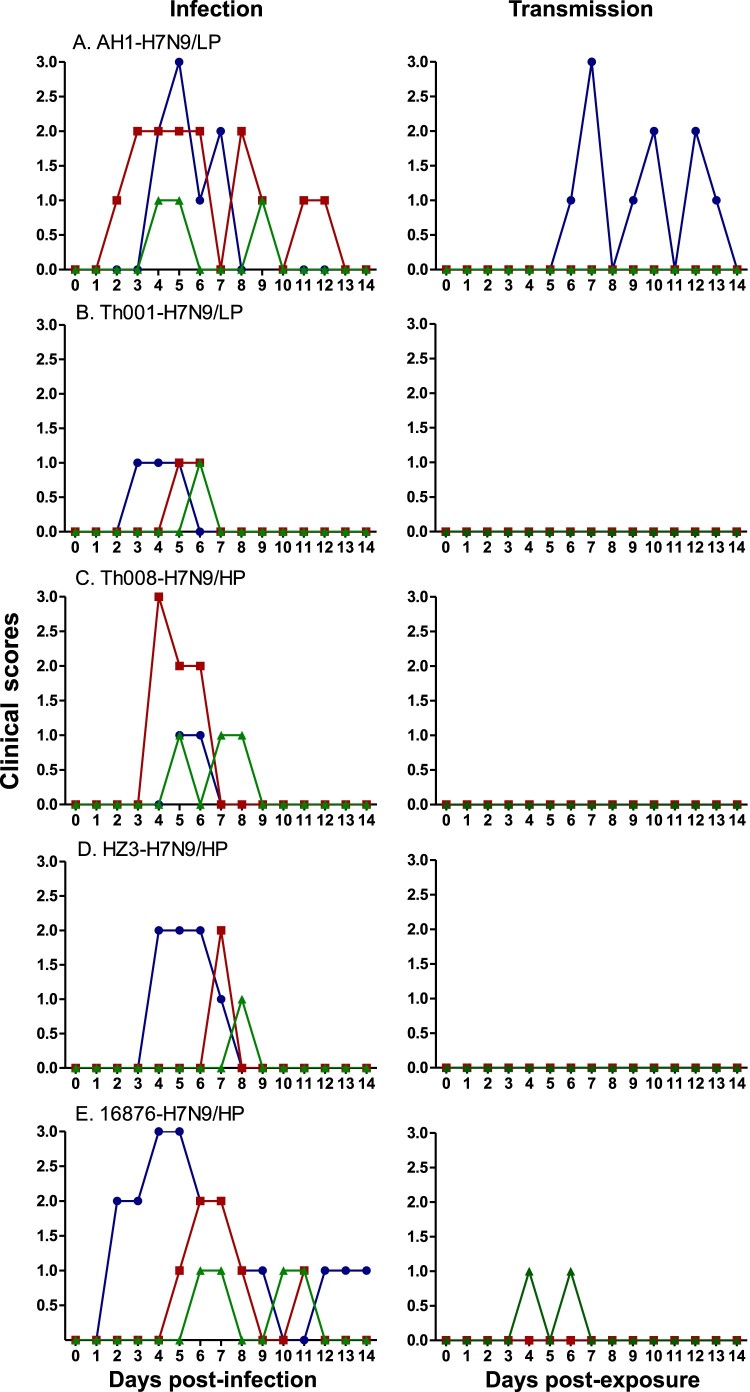
Clinical scores of the H7N9-infected and exposed ferrets. Clinical symptoms of ferrets in the infected (*n* = 3) and transmission (*n* = 3) groups were monitored and recorded daily. Scores were the joint compilation of both parts as described by the Reuman scale.

Nasal congestion, sneezing, discharge on external nasal cavities and mouth breathing were observed in all infected ferrets, but animals infected with different H7N9 isolates had variable illnesses in terms of length and disease severity. The average duration and standard deviation of disease symptoms for A/Anhui/1/2013 (LP) ferrets was 7.0 ± 3.6 days, with average peak clinical scores of 2.0 ± 1.0. For A/Chicken/Heyuan/16876/2016 (HP), the average disease duration was 8.7 ± 3.8 days and clinical scores were 2.0 ± 1.0. Ferrets infected with A/Guangdong/Th008/2017 (HP) showed moderate illness, with a disease duration of 3.0 ± 1.0 days and clinical scores of 1.7 ± 1.2. A/Chicken/Huizhou/HZ-3/2017 (HP) animals displayed disease symptoms for 2.0 ± 1.7 days and scores of 1.7 ± 0.6, and A/Shenzhen/Th001/2016 (LP) was 2.0 ± 1.0 days with scores of 1.0 ± 0.0, which was the mildest of all tested strains (Figure 2 and Table 1).

**Figure 3.**
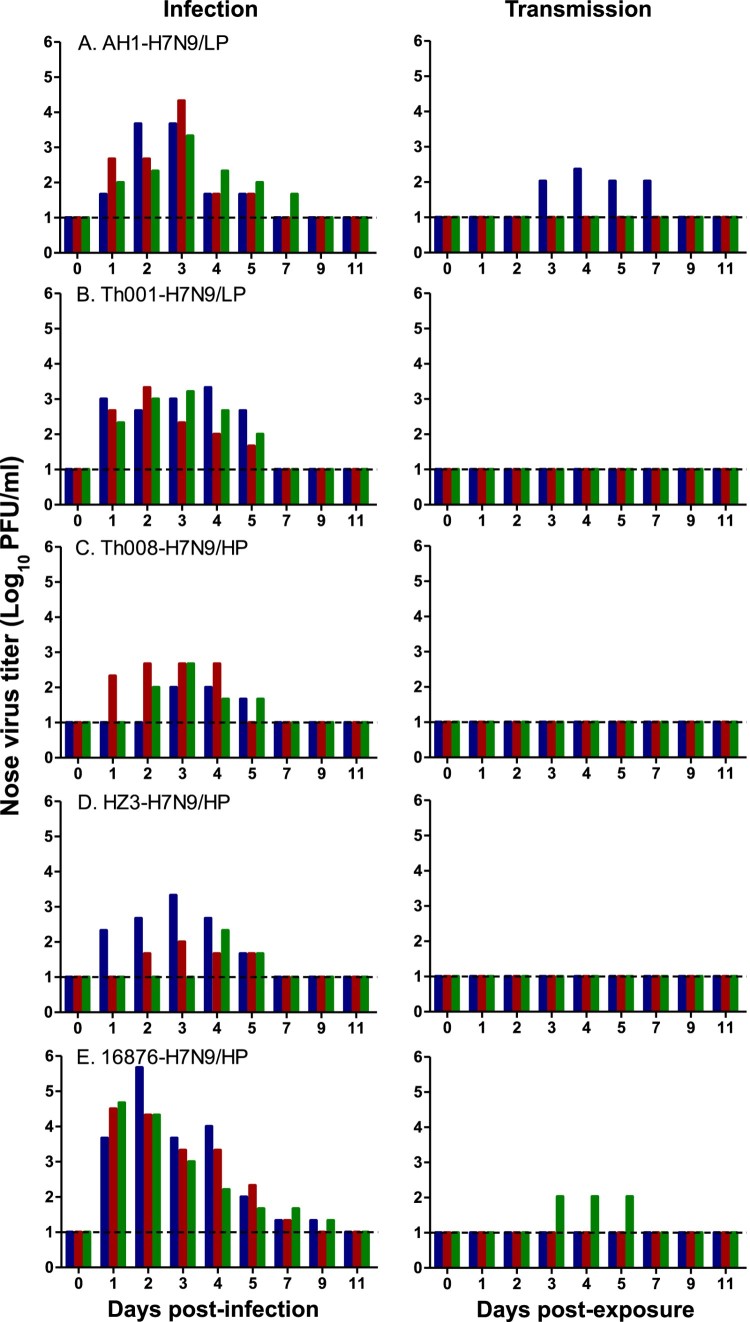
Virus titres in the nasal cavities of H7N9-infected and exposed ferrets. Ferrets were inoculated with 10^6^ EID_50_ of the indicated H7N9 isolate, and housed at 1-day post-infection (d.p.i.) with a naive animal in a neighbouring cage – in which direct contact between the animals is not possible. Nasal swabs were collected at 1–7, 9, 11 d.p.i., and 1–7, 9, 11 days post-exposure (d.p.e.) from ferrets in each infection (*n* = 3) and exposure (*n* = 3) group.

Nasal and throat swabs were collected from inoculated animals at 1–7, 9 and 11 days post-infection (d.p.i.) to quantitate virus titres. Average peak virus titres in the nasal cavity were the highest and duration of virus shedding was the longest in the A/Chicken/Heyuan/16876/2016 (HP) group (1.8E5 ± 2.5E5 plaque forming units (PFU)/ml and 8.3 ± 1.2 days), followed by A/Anhui/1/2013 (LP) (9.4E3 ± 1.0E4 PFU/ml and 5.7 ± 1.2 days), A/Shenzhen/Th001/2016 (LP) (2.0E3 ± 3.0E2 PFU/ml and 5.0 ± 0.0 days), A/Chicken/Huizhou/HZ-3/2017 (HP) (8.2E2 ± 1.1E3 PFU/ml and 3.7 ± 1.5 days), and A/Guangdong/Th008/2017 (HP) (3.5E2 ± 2.1E2 PFU/ml and 3.7 ± 0.6 days) (Figures 3, S2 and Table 1).

### Transmissibility in ferrets

To investigate viral transmissibility, groups of ferrets (*n* = 3) were infected intranasally (i.n.) with one of the aforementioned isolates at a dose of 10^6^ 50% egg infective dose (EID_50_). The inoculated animals were then housed individually with a naïve ferret in a neighbouring cage at 1 d.p.i. Clinical symptoms, virus shedding and seroconversion of the exposed ferrets in each group were monitored. It can be seen that animals in the A/Anhui/1/2013 (LP) and A/Chicken/Heyuan/16876/2016 (HP) groups showed weight loss (Figure S1), whereas ferrets in the other groups did not lose weight. All challenged animals were found to have seroconverted with haemagglutinin inhibition (HI) titres in the range of 80–5000 (Figure S3A).

In the exposed ferrets, virus shedding from the nose and throat was detected in one out of three animals infected with A/Anhui/1/2013 (LP) and A/Chicken/Heyuan/16876/2016 (HP), but not in the other groups (Figures 3 and S2). Additionally, one out of three ferrets in the A/Chicken/Heyuan/16876/2016 (HP) group was found to have seroconverted with an HI titre of 80, whereas two out of three ferrets in the A/Anhui/1/2013 (LP) group were seropositive with mean HI titres of 120. All animals from the other transmission groups were seronegative (HI ≤ 20) (Figure S3B and Table 1).

## Discussion

When H7N9 first emerged in 2013, it was considered an unusually dangerous virus because H7N9 can be more easily transmitted from poultry to humans compared to other AIVs. However, comprehensive prevention and control measures, such as strengthening the management of live poultry markets [16] as well as poultry vaccinations after Wave 5, has resulted in curtailed numbers of human cases [17]. The ability to bind both avian- and human-origin receptors of the H7N9 virus was identified as one critical factor for human infections [10–12,18]. However, H7N9 retains a preference for avian-type receptors, which may restrict effective human-to-human transmission. Residues V186 and L226 on HA were considered to be the pivotal amino acid residues for binding avidity to human receptors [13,18].

There are at least two barriers for cross-species transmission “host jump” of AIV to humans or mammals [19], and were studied for H7N9 from previous infection waves [10–12,18,20–23]. The first barrier is the ability to bind human-origin sialic acid receptors, which facilitates virus entry into human cells [19]. Based on a recent study on H7N9 AIV from Wave 5, nearly all HP-H7N9 viruses, including those tested in the present study, possessed residue Q226 [2] for binding avian-origin receptors [13]. While A/Chicken/Heyuan/16876/2016 (HP), A/Chicken/Huizhou/HZ-3/2017 (HP) and A/Guangdong/Th008/2017 (HP) possess a similar affinity for avian-receptors, but they have different binding abilities to human-origin receptors. This may be because residues 171–172 near the receptor binding sites on HA1 are different for the three isolates (KE, A/Guangdong/Th008/2017 (HP)), (RK, A/Chicken/Huizhou/HZ-3/2017 (HP)) and (RE, A/Chicken/Heyuan/16876/2016 (HP)). Interestingly, we also found that A/Shenzhen/Th001/2016 (LP) preferred to bind human-type receptors with a similar affinity compared to the precursor A/Anhui/1/2013 (LP), and a substantially lower avidity to avian-type receptors (Figure 1). The diverse receptor binding abilities and the binding preference of some H7N9 isolates towards human-type receptors means further monitoring of this virus is warranted.

Additionally, we found that the Wave 5 H7N9 isolates behaved differently in ferrets. While A/Chicken/Heyuan/16876/2016 (HP) was similar to the precursor A/Anhui/1/2013 (LP) from Wave 1, infection of ferrets with other Wave 5 H7N9 isolates resulted in milder disease. This is evidenced and supported by several parameters, including the length of disease symptoms, clinical score, peak viral titres after infection, and length of virus shedding. It was shown that ferrets infected with A/Chicken/Heyuan/16876/2016 (HP) and A/Anhui/1/2013 (LP) resulted in virus transmission to naive ferrets and a higher rate of seroconversion, consistent with the idea that sicker ferrets had a higher probability to transmit the virus to naive animals. In addition, A/Guangdong/Th008/2017 (HP) showed low replication ability and could not transmit in ferrets. The virus may possibly be inhibited by the NAI-resistant mutation R292 K (N2 numbering) in its NA protein, as suggested by a previous study [24]. In the future, it will be important to complement and strengthen these observations with more *in vitro* and *in vivo* data, such as replication kinetics of the various HP- and LP-H7N9 isolates in susceptible cell lines, as well as tissue collection from infected ferrets during scheduled necropsy.

It should be noted that several other studies investigating the pathogenicity and transmissibility of HP-H7N9 isolates were published recently. In one study, the authors demonstrated that their Wave 5 HP-H7N9 isolate was more pathogenic in ferrets compared to LP-H7N9 with evidence of transmission via respiratory droplets [14]. A second study showed that their Wave 5 HP-H7N9 isolate was initially not lethal to mice and ferrets, but after replication in ferrets the passaged acquired mutations that resulted in enhanced virulence and transmissibility in these animals via respiratory droplet [15]. In a third study, the authors found that their HP-H7N9 isolates were more virulent compared to LP-H7N9 viruses in the mouse and ferret animal models, with enhanced tropism for brain tissue, but that their isolates did not transmit well via respiratory droplets [25]. These results, combined with ours, strongly suggest that the pathogenicity and transmissibility of circulating H7N9 viruses is isolate-specific, and that testing of multiple wild-type H7N9 isolates in the future will be important for providing a full picture of the exact public health threat posed by these viruses.

## Materials and methods

### Viruses and cells

LP- and HP-H7N9 AIVs, including: A/Shenzhen/Th001/2016, A/Guangdong/Th008/2017, A/Chicken/Heyuan/16876/2016, A/Chicken/Huizhou/HZ-3/2017 were isolated from either humans or poultry during Wave 5 [2,7,8]. Except for A/Shenzhen/Th001/2016, which belongs to the Pearl River Delta lineage, the other isolates are all from the Yangtze River Delta lineage. Two isolates (A/Shenzhen/Th001/2016, A/Guangdong/Th008/2016) caused severe disease in humans, but all patients recovered after treatment. The ancestor reference strain A/Anhui/1/2013[5] was isolated from Wave 1 during 2013. Stock viruses were propagated in the allantoic cavities of 10-day-old specific pathogen-free (SPF) embryonated chicken eggs. MDCK cells (ATCC) were cultured at 37°C with 5% CO_2_, in Dulbecco’s modified Eagle’s medium (DMEM, Invitrogen) containing 10% fetal bovine serum (FBS, Gibco), 100 IU/ml penicillin and streptomycin.

### Receptor-binding assay

Receptor-binding specificity was determined using the solid-phase direct binding assay, as described previously [26].

### Animals

Castrated ferrets (*Mustela putorius furo*), 4–6 months old, were available from the in-house colony. Animals were shown to be serologically negative by HI assay against the following influenza viruses: A/California/07/2009(H1N1), A/Brisbane/10/2007(H3N2), A/Shenzhen/Th002/2016(H5N6), A/Brambling/Beijing/16/2012(H9N2) and the H7N9 viruses used in this study. Animals were housed in standard isolator cages and fed food and water *ad libitum*.

### Pathogenicity in ferrets

To determine the pathogenicity of HP- and LP-H7N9, groups of three ferrets were inoculated i.n. with 10^6^ EID_50_/500 μl of AH1-H7N9/LP, Th001-H7N9/LP, Th008-H7N9/HP, HZ3-H7N9/HP or 16876-H7N9/HP. Clinical signs of the infected animals were observed daily. The overall clinical score was the sum of two parts, as described by a previous report [27]. Part one is nasal symptoms: no symptoms (0), nasal rattling or sneezing (1), nasal discharge on external nasal cavity (2) and mouth breathing (3). Part 2 is activity level: playful (0), not initiating play (1), alert but not playful (2) and not playful, not alert (3). Nasal and throat swabs of the infected ferrets were collected from inoculated animals at 1–7, 9, and 11 d.p.i. and transferred to 0.5 ml of phosphate-buffered saline (PBS) for virus titration.

### Transmissibility in ferrets

For transmission experiments, three ferrets from each group were inoculated with 10^6^ EID_50_ of the Waves 1 and 5 H7N9 viruses. Transmission experiments were conducted in cages as previously described [5], designed at a distance of ∼8 centimeters (cm) to prevent any direct contact between animals, but to allow airflow from an inoculated ferret to a neighbouring naïve animal.

At 1 d.p.i., inoculated animals were housed individually with a naïve ferret in a neighbouring cage. All items that came into contact with the ferrets or the bedding were decontaminated in order to prevent inadvertent physical transmission of the virus by the investigators. The ferrets were observed for clinical signs daily as an indicator of disease. Nasal and throat swabs were collected from naïve animals at 1–7, 9, 11, 14 d.p.e. Virus titres were determined by plaque assay in MDCK cells. Post-exposure sera were collected from inoculated animals at 21 d.p.i. or exposed animals at 21 d.p.e. to test for seroconversion by HI assay.

### Haemagglutination inhibition (HI) antibody test

Prior to the HI test, the sera of ferrets in the infected and exposed groups were treated by receptor-destroying enzyme (RDE) at 37°C for 16 h, incubated at 56°C for 0.5 h, and treated with 25% chicken red blood cells (CRBC). The HI test for the RDE-treated sera was determined with 1% CRBC following the method described in the WHO Manual on Animal Influenza Diagnosis and Surveillance (http://www.who.int/csr/resources/publications/influenza/whocdscsrncs20025rev.pdf). HI titres ≥ 20 were considered as positive for H7N9 virus.

### Virus titrations

Stock viruses were titrated by haemagglutination (HA) assay with 1% CRBC, as well as by EID_50_ in SPF chicken embryos, according to previously described methods [20,28,29]. Briefly, 10-fold serial dilutions of the viruses were used to inoculate chicken embryos at 37°C for 72 h. The EID_50_ values were calculated by the Reed and Muench method.

Nasal and throat swabs from the infected and exposed ferrets were suspended in 0.5 ml of PBS, and the supernatant collected for 10-fold serial dilutions in PBS. The dilutions were inoculated in MDCK cells for one hour, washed three times with PBS, and overlaid with 2 ml of DMEM containing 1% (wt/vol) low-melting-point agarose, 2 μg/ml TPCK-treated trypsin, 100 IU/ml penicillin and streptomycin. Viral titres were counted as PFU at 3 d.p.i.

### Biosafety and ethics

This study was approved by the Ethics Committee of Institute of Laboratory Animal Science (ILAS) at the Chinese Academy of Medical Sciences & Peking Union Medical College (PUMC) (BLL17005) and Ethics Committee of Institue of Microbilogy, Chinese Academy of Sciences (SQIMCAS2016016). All efforts were made to minimize animal suffering and to use the minimum number of animals required to reach the conclusions of this study. All experiments with live H7N9 viruses were performed in the biosafety level 3 (BSL-3) laboratory.

### Statistical analysis

Differences between two groups were analysed by two-tailed Student’s *t*-test. A probability (*P*) value of <.05 was considered as statistically significant (*P < .05; **P < .01; ***P < .001).
